# (*E*)-1-(2,4-Dinitro­phen­yl)-2-[1-(3-fluoro­phen­yl)ethyl­idene]hydrazine

**DOI:** 10.1107/S160053681201937X

**Published:** 2012-05-05

**Authors:** Suchada Chantrapromma, Boonlerd Nilwanna, Thawanrat Kobkeatthawin, Patcharaporn Jansrisewangwong, Hoong-Kun Fun

**Affiliations:** a Crystal Materials Research Unit, Department of Chemistry, Faculty of Science, Prince of Songkla University, Hat-Yai, Songkhla 90112, Thailand; b X-ray Crystallography Unit, School of Physics, Universiti Sains Malaysia, 11800 USM, Penang, Malaysia

## Abstract

The mol­ecule of the title hydrazone derivative, C_14_H_11_FN_4_O_4_, is nearly planar, with a dihedral angle between the benzene rings of 3.71 (7)°. The central ethyl­idenehydrazine N—N=C—C plane makes dihedral angles of 5.32 (10) and 9.02 (10)° with the 2,4-dinitro- and 3-fluoro-substituted benzene rings, respectively. An intra­molecular N—H⋯O bond generates an *S*(6) ring motif. In the crystal, mol­ecules are linked by weak C—H⋯O inter­actions into a sheet parallel to (10-1). The mol­ecules are further stacked along the *a* axis by π–π inter­actions with centroid–centroid distances of 3.6314 (9) and 3.7567 (10) Å. A C⋯F short contact [2.842 (3) Å] is observed. The 3-fluoro­phenyl group is disordered over two orientations with a site-occupancy ratio of 0.636 (3):0.364 (3).

## Related literature

For bond-length data, see: Allen *et al.* (1987 ▶). For hydrogen-bond motifs, see: Bernstein *et al.* (1995 ▶). For related structures, see: Chantrapromma *et al.* (2011 ▶); Fun *et al.* (2011 ▶, 2012 ▶); Nilwanna *et al.* (2011 ▶). For background to and the biological activity of hydro­zones, see: Cui *et al.* (2010 ▶); Gokce *et al.* (2009 ▶); Krishnamoorthy *et al.* (2011 ▶); Molyneux (2004 ▶); Wang *et al.* (2009 ▶). For the stability of the temperature controller used in the data collection, see: Cosier & Glazer (1986 ▶).
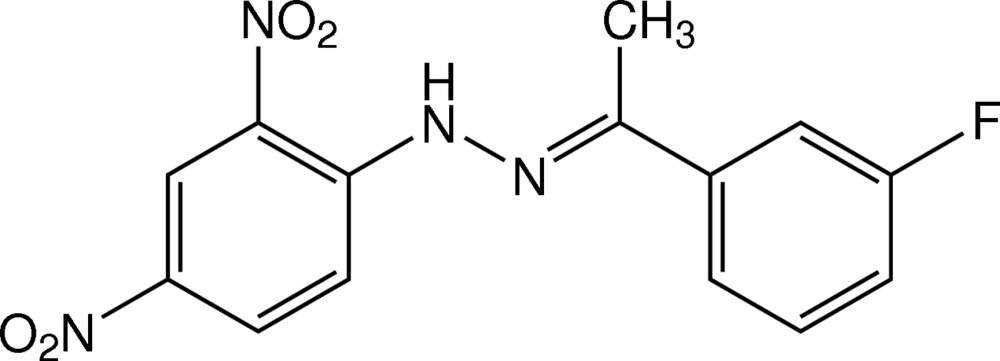


## Experimental

### 

#### Crystal data

C_14_H_11_FN_4_O_4_
*M* *_r_* = 318.27
Monoclinic, 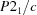

*a* = 7.0165 (6) Å
*b* = 13.3336 (11) Å
*c* = 14.4498 (12) Å
β = 94.791 (2)°
*V* = 1347.1 (2) Å^3^
*Z* = 4
Mo *K*α radiation
μ = 0.13 mm^−1^
*T* = 100 K
0.39 × 0.15 × 0.14 mm

#### Data collection

Bruker APEX DUO CCD area-detector diffractometer
Absorption correction: multi-scan (*SADABS*; Bruker, 2009 ▶) *T*_min_ = 0.952, *T*_max_ = 0.982
15079 measured reflections
3874 independent reflections
3126 reflections with *I* > 2σ(*I*)
*R*_int_ = 0.054

#### Refinement

*R*[*F* ^2^ > 2σ(*F* ^2^)] = 0.052
*wR*(*F* ^2^) = 0.155
*S* = 1.07
3874 reflections
223 parameters
2 restraints
H atoms treated by a mixture of independent and constrained refinement
Δρ_max_ = 0.70 e Å^−3^
Δρ_min_ = −0.59 e Å^−3^

### 

Data collection: *APEX2* (Bruker, 2009 ▶); cell refinement: *SAINT* (Bruker, 2009 ▶); data reduction: *SAINT*; program(s) used to solve structure: *SHELXTL* (Sheldrick, 2008 ▶); program(s) used to refine structure: *SHELXTL*; molecular graphics: *SHELXTL*; software used to prepare material for publication: *SHELXTL* and *PLATON* (Spek, 2009 ▶).

## Supplementary Material

Crystal structure: contains datablock(s) global, I. DOI: 10.1107/S160053681201937X/is5116sup1.cif

Structure factors: contains datablock(s) I. DOI: 10.1107/S160053681201937X/is5116Isup2.hkl

Supplementary material file. DOI: 10.1107/S160053681201937X/is5116Isup3.cml

Additional supplementary materials:  crystallographic information; 3D view; checkCIF report

## Figures and Tables

**Table 1:** Hydrogen-bond geometry (Å, °)

*D*—H⋯*A*	*D*—H	H⋯*A*	*D*⋯*A*	*D*—H⋯*A*
N2—H1*N*2⋯O1	0.89 (2)	1.90 (2)	2.6038 (18)	135 (2)
C9—H9*A*⋯O1^i^	0.93	2.58	3.413 (2)	150
C13—H13*A*⋯O4^ii^	0.93	2.44	3.176 (2)	137

Symmetry codes: (i) 
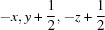
; (ii) 
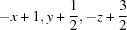
.

**Fig. 1.**
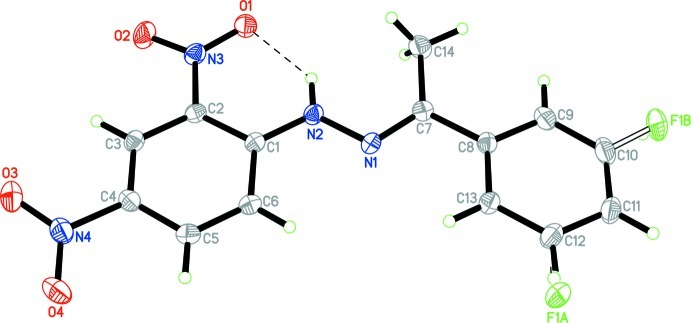
The molecular structure of the title compound, with 45% probability displacement ellipsoids and the atom-numbering scheme. Open bonds show the minor component. The hydrogen bond is shown as a dashed line.

**Fig. 2.**
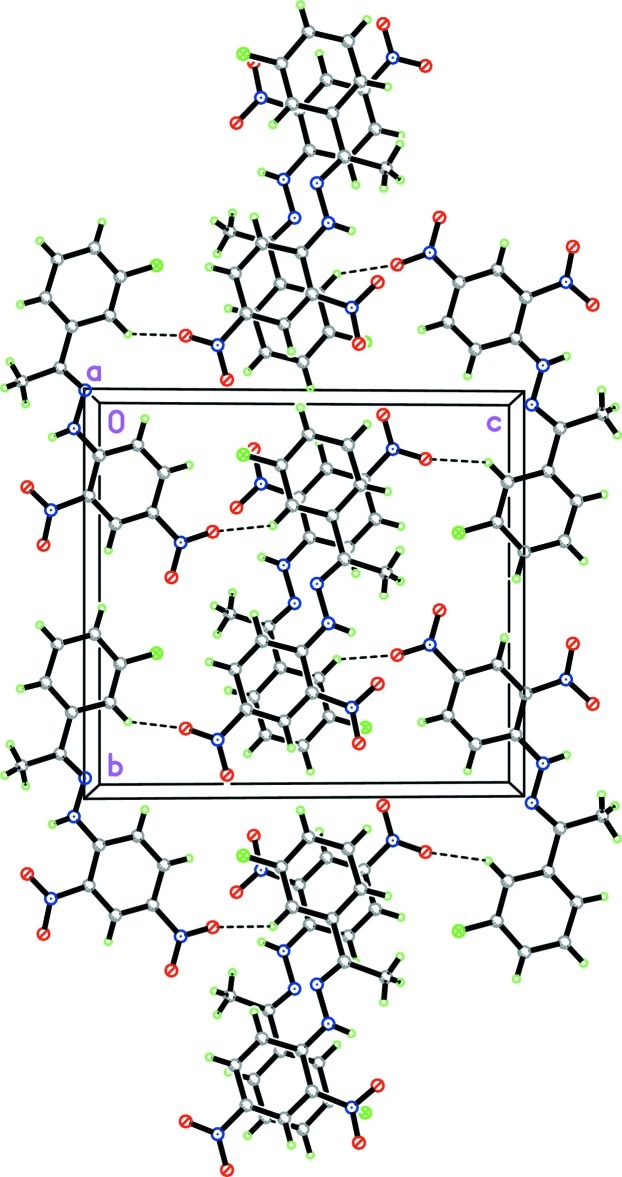
The crystal packing of the major component of the title compound viewed down the a axis. Hydrogen bonds are shown as dashed lines.

## supplementary crystallographic information

### Comment

The variety of interesting biological activities of hydrazones and their complexes such as antibacterial, antifungal, anti-inflammatory as well as antioxidant properties (Cui *et al.*, 2010; Gokce *et al.*, 2009; Krishnamoorthy *et al.*, 2011; Wang *et al.*, 2009) has prompted us to synthesize several hydrazone derivatives and to study for their biological activities. However the title compound (I) which was synthesized for the evaluation of its antioxidant activity by DPPH scavenging (Molyneux, 2004) was found to be inactive. Herein we report the synthesis and crystal structure of (I).

In the molecular structure of (I), C_14_H_11_FN_4_O_4_, the F atoms of the 3-fluorophenyl group is disordered over two positions with the major component *A* and the minor *B* component rotated by 180° about the C7—C8 bond and having a refined site-occupancy ratio of 0.636 (3):0.364 (3) (Fig. 1). The molecule is nearly planar with a dihedral angle between the two benzene rings being 3.71 (7)°. The middle ethylidenehydrazine bridge is planar with the torsion angle N2–N1–C7–C14 = 0.8 (2)°. The mean plane through this middle bridge makes dihedral angles of 5.32 (10) and 9.02 (10)° with the 2,4-dinitrophenyl and 3-fluorophenyl rings, respectively. The two nitro groups of the 2,4-dinitrophenyl unit are slightly twisted with the attached benzene ring as indicated by the torsion angles O1–N3–C2–C1 = -3.8 (2)°, O2–N3–C2–C1 = 176.52 (15)°, O3–N4–C4–C3 = -8.3 (2)° and O4–N4–C4–C3 = 171.96 (16)°. An intramolecular N2—H1N2···O1 hydrogen bond (Fig.1 and Table 1) generates an S(6) ring motif (Bernstein *et al.*, 1995). The bond distances are in normal ranges (Allen *et al.*, 1987) and are comparable with the closely related structures (Chantrapromma *et al.*, 2011; Fun *et al.*, 2011, 2012; Nilwanna *et al.*, 2011).

In the crystal packing (Fig. 2), the molecules are linked by weak C—H···O interactions (Table 1) into a sheet parallel to the (101) plane and these sheets are stacked along the *a* axis by π–π interactions with centroid-to-centroid distances *Cg*1···*Cg*2 = 3.7567 (10)^iii^ and 3.6314 (9)^iv^ Å [symmetry codes (iii) = -x, 1-y, 1-z and (iv) = 1-x, 1-y, 1-z)]. A C14···F1B^v^ [2.905 (3) Å ; symmetry code (v) = -x, -1/2+y, 1/2-z] short contact is observed.

### Experimental

The title compound (I) was synthesized by dissolving 2,4-dinitrophenylhydrazine (0.40 g, 2 mmol) in ethanol (10.00 ml) and H_2_SO_4_ (conc.) (98 %, 0.50 ml) was slowly added with stirring. 3-Fluoroacetophenone (0.25 ml, 2 mmol) was then added to the solution with continuous stirring. The solution was stirred for 1 hr yielding an yellow solid, which was filtered off and washed with methanol. Yellow block-shaped single crystals of the title compound suitable for *x*-ray structure determination were recrystallized from ethanol by slow evaporation of the solvent at room temperature over several days. M.p. 503-504 K.

### Refinement

Amide H atom was located in a Fourier difference map and refined isotropically. The remaining H atoms were positioned geometrically and allowed to ride on their parent atoms, with d(C—H) = 0.93 Å for aromatic and 0.96 Å for CH_3_ atoms. The *U*_iso_ values were constrained to be 1.5*U*_eq_ of the carrier atom for methyl H atoms and 1.2*U*_eq_ for the remaining H atoms. A rotating group model was used for the methyl groups. The F atom was found to be disordered over two sites in a 0.636 (3): 0.364 (3) occupancy ratio. In the final refinement, distance restraints were used for the disordered C—F bonds.

### Crystal data

**Table:**

C_14_H_11_FN_4_O_4_	*F*(000) = 656
*M**_r_* = 318.27	*D*_x_ = 1.569 Mg m^−^^3^
Monoclinic, *P*2_1_/*c*	Melting point = 503–504 K
Hall symbol: -P 2ybc	Mo *K*α radiation, λ = 0.71073 Å
*a* = 7.0165 (6) Å	Cell parameters from 3874 reflections
*b* = 13.3336 (11) Å	θ = 2.1–30.0°
*c* = 14.4498 (12) Å	µ = 0.13 mm^−^^1^
β = 94.791 (2)°	*T* = 100 K
*V* = 1347.1 (2) Å^3^	Block, yellow
*Z* = 4	0.39 × 0.15 × 0.14 mm

### Data collection

**Table:**

Bruker APEX DUO CCD area-detector diffractometer	3874 independent reflections
Radiation source: sealed tube	3126 reflections with *I* > 2σ(*I*)
Graphite monochromator	*R*_int_ = 0.054
φ and ω scans	θ_max_ = 30.0°, θ_min_ = 2.1°
Absorption correction: multi-scan (*SADABS*; Bruker, 2009)	*h* = −9→9
*T*_min_ = 0.952, *T*_max_ = 0.982	*k* = −18→18
15079 measured reflections	*l* = −20→19

### Refinement

**Table:**

Refinement on *F*^2^	Primary atom site location: structure-invariant direct methods
Least-squares matrix: full	Secondary atom site location: difference Fourier map
*R*[*F*^2^ > 2σ(*F*^2^)] = 0.052	Hydrogen site location: inferred from neighbouring sites
*wR*(*F*^2^) = 0.155	H atoms treated by a mixture of independent and constrained refinement
*S* = 1.07	*w* = 1/[σ^2^(*F*_o_^2^) + (0.0567*P*)^2^ + 0.806*P*] where *P* = (*F*_o_^2^ + 2*F*_c_^2^)/3
3874 reflections	(Δ/σ)_max_ = 0.001
223 parameters	Δρ_max_ = 0.70 e Å^−^^3^
2 restraints	Δρ_min_ = −0.59 e Å^−^^3^

### Special details

**Table:**

Experimental. The crystal was placed in the cold stream of an Oxford Cryosystems Cobra open-flow nitrogen cryostat (Cosier & Glazer, 1986) operating at 100.0 (1) K.
Geometry. All esds (except the esd in the dihedral angle between two l.s. planes) are estimated using the full covariance matrix. The cell esds are taken into account individually in the estimation of esds in distances, angles and torsion angles; correlations between esds in cell parameters are only used when they are defined by crystal symmetry. An approximate (isotropic) treatment of cell esds is used for estimating esds involving l.s. planes.
Refinement. Refinement of F^2^ against ALL reflections. The weighted R-factor wR and goodness of fit S are based on F^2^, conventional R-factors R are based on F, with F set to zero for negative F^2^. The threshold expression of F^2^ > 2sigma(F^2^) is used only for calculating R-factors(gt) etc. and is not relevant to the choice of reflections for refinement. R-factors based on F^2^ are statistically about twice as large as those based on F, and R- factors based on ALL data will be even larger.

### Fractional atomic coordinates and isotropic or equivalent isotropic displacement parameters (Å^2^)

**Table:**

	*x*	*y*	*z*	*U*_iso_*/*U*_eq_	Occ. (<1)
O1	0.16769 (19)	0.27587 (9)	0.33374 (8)	0.0286 (3)	
O2	0.2526 (2)	0.12572 (9)	0.37606 (9)	0.0362 (3)	
O3	0.4548 (2)	0.04192 (10)	0.68409 (10)	0.0395 (3)	
O4	0.5525 (2)	0.15695 (12)	0.78252 (10)	0.0421 (4)	
N1	0.22627 (19)	0.52482 (9)	0.47367 (9)	0.0204 (3)	
N2	0.2316 (2)	0.42615 (10)	0.44717 (9)	0.0211 (3)	
H1N2	0.187 (4)	0.4037 (18)	0.3918 (17)	0.039 (6)*	
N3	0.2368 (2)	0.21543 (10)	0.39303 (9)	0.0239 (3)	
N4	0.4801 (2)	0.13015 (11)	0.70636 (10)	0.0283 (3)	
C1	0.2965 (2)	0.35508 (11)	0.50920 (10)	0.0190 (3)	
C2	0.2999 (2)	0.25164 (11)	0.48499 (10)	0.0197 (3)	
C3	0.3613 (2)	0.17866 (11)	0.54990 (11)	0.0212 (3)	
H3A	0.3624	0.1113	0.5332	0.025*	
C4	0.4199 (2)	0.20773 (12)	0.63873 (11)	0.0224 (3)	
C5	0.4229 (2)	0.30902 (13)	0.66526 (11)	0.0231 (3)	
H5A	0.4656	0.3272	0.7256	0.028*	
C6	0.3626 (2)	0.38075 (12)	0.60177 (10)	0.0210 (3)	
H6A	0.3649	0.4478	0.6196	0.025*	
C7	0.1518 (2)	0.58571 (11)	0.41102 (10)	0.0202 (3)	
C8	0.1511 (2)	0.69296 (11)	0.43865 (11)	0.0205 (3)	
C9	0.1014 (2)	0.76901 (13)	0.37412 (12)	0.0264 (3)	
H9A	0.0644	0.7534	0.3125	0.032*	
C10	0.1085 (3)	0.86754 (13)	0.40379 (13)	0.0332 (4)	
H10A	0.0755	0.9175	0.3605	0.040*	0.636 (4)
F1A	0.2507 (3)	0.84685 (12)	0.64414 (10)	0.0370 (5)	0.636 (4)
C11	0.1615 (3)	0.89560 (13)	0.49351 (14)	0.0310 (4)	
H11A	0.1654	0.9626	0.5115	0.037*	
C12	0.2086 (2)	0.81953 (12)	0.55571 (10)	0.0277 (3)	
H12A	0.2449	0.8363	0.6171	0.033*	0.364 (4)
F1B	0.0366 (5)	0.9380 (2)	0.3455 (2)	0.0441 (10)	0.364 (4)
C13	0.2046 (2)	0.71958 (11)	0.53113 (11)	0.0227 (3)	
H13A	0.2369	0.6704	0.5753	0.027*	
C14	0.0737 (3)	0.55223 (14)	0.31617 (11)	0.0284 (4)	
H14A	−0.0124	0.4970	0.3219	0.043*	
H14B	0.1771	0.5314	0.2811	0.043*	
H14C	0.0064	0.6068	0.2848	0.043*	

### Atomic displacement parameters (Å^2^)

**Table:**

	*U*^11^	*U*^22^	*U*^33^	*U*^12^	*U*^13^	*U*^23^
O1	0.0396 (7)	0.0257 (6)	0.0197 (5)	0.0008 (5)	−0.0015 (5)	−0.0019 (4)
O2	0.0563 (9)	0.0211 (6)	0.0306 (6)	0.0018 (6)	0.0001 (6)	−0.0088 (5)
O3	0.0511 (9)	0.0265 (6)	0.0403 (7)	−0.0014 (6)	−0.0006 (6)	0.0095 (5)
O4	0.0501 (9)	0.0426 (8)	0.0307 (7)	0.0046 (7)	−0.0129 (6)	0.0045 (6)
N1	0.0226 (6)	0.0174 (6)	0.0214 (6)	−0.0015 (5)	0.0035 (5)	−0.0011 (5)
N2	0.0264 (7)	0.0184 (6)	0.0183 (6)	−0.0002 (5)	0.0016 (5)	−0.0019 (4)
N3	0.0285 (7)	0.0220 (6)	0.0212 (6)	−0.0015 (5)	0.0027 (5)	−0.0042 (5)
N4	0.0263 (7)	0.0300 (7)	0.0285 (7)	0.0008 (6)	0.0015 (6)	0.0062 (6)
C1	0.0179 (6)	0.0197 (6)	0.0198 (6)	−0.0015 (5)	0.0037 (5)	−0.0013 (5)
C2	0.0191 (7)	0.0213 (7)	0.0188 (6)	−0.0017 (5)	0.0029 (5)	−0.0025 (5)
C3	0.0196 (7)	0.0195 (6)	0.0248 (7)	−0.0017 (5)	0.0034 (5)	−0.0002 (5)
C4	0.0192 (7)	0.0257 (7)	0.0223 (7)	−0.0008 (6)	0.0019 (5)	0.0039 (6)
C5	0.0205 (7)	0.0279 (8)	0.0207 (7)	−0.0027 (6)	0.0014 (5)	−0.0017 (5)
C6	0.0206 (7)	0.0218 (7)	0.0207 (7)	−0.0019 (5)	0.0013 (5)	−0.0033 (5)
C7	0.0197 (7)	0.0216 (7)	0.0197 (6)	0.0008 (5)	0.0037 (5)	−0.0017 (5)
C8	0.0184 (7)	0.0201 (7)	0.0233 (7)	0.0005 (5)	0.0029 (5)	0.0011 (5)
C9	0.0234 (7)	0.0276 (8)	0.0278 (8)	0.0030 (6)	0.0003 (6)	0.0044 (6)
C10	0.0272 (8)	0.0261 (8)	0.0454 (10)	0.0061 (7)	−0.0008 (7)	0.0070 (7)
F1A	0.0497 (11)	0.0250 (8)	0.0343 (9)	0.0030 (7)	−0.0078 (8)	−0.0097 (6)
C11	0.0257 (8)	0.0192 (7)	0.0478 (10)	0.0025 (6)	0.0020 (7)	−0.0024 (7)
C12	0.0258 (8)	0.0222 (7)	0.0353 (9)	−0.0019 (6)	0.0031 (7)	−0.0064 (6)
F1B	0.059 (2)	0.0236 (15)	0.048 (2)	0.0038 (14)	−0.0056 (16)	0.0141 (13)
C13	0.0247 (7)	0.0192 (7)	0.0241 (7)	−0.0009 (6)	0.0023 (6)	−0.0003 (5)
C14	0.0348 (9)	0.0297 (8)	0.0203 (7)	0.0060 (7)	−0.0011 (6)	−0.0033 (6)

### Geometric parameters (Å, º)

**Table:**

O1—N3	1.2447 (18)	C7—C8	1.485 (2)
O2—N3	1.2279 (18)	C7—C14	1.501 (2)
O3—N4	1.229 (2)	C8—C9	1.402 (2)
O4—N4	1.226 (2)	C8—C13	1.403 (2)
N1—C7	1.293 (2)	C9—C10	1.381 (3)
N1—N2	1.3716 (18)	C9—H9A	0.9300
N2—C1	1.357 (2)	C10—F1B	1.3322 (10)
N2—H1N2	0.89 (2)	C10—C11	1.371 (3)
N3—C2	1.4478 (19)	C10—H10A	0.9300
N4—C4	1.461 (2)	F1A—C12	1.3377 (10)
C1—C6	1.420 (2)	C11—C12	1.377 (2)
C1—C2	1.424 (2)	C11—H11A	0.9300
C2—C3	1.395 (2)	C12—C13	1.379 (2)
C3—C4	1.371 (2)	C12—H12A	0.9300
C3—H3A	0.9300	C13—H13A	0.9300
C4—C5	1.404 (2)	C14—H14A	0.9600
C5—C6	1.368 (2)	C14—H14B	0.9600
C5—H5A	0.9300	C14—H14C	0.9600
C6—H6A	0.9300		
			
C7—N1—N2	115.18 (13)	C8—C7—C14	121.53 (14)
C1—N2—N1	120.07 (13)	C9—C8—C13	118.84 (15)
C1—N2—H1N2	115.7 (16)	C9—C8—C7	121.61 (14)
N1—N2—H1N2	124.1 (16)	C13—C8—C7	119.55 (13)
O2—N3—O1	121.97 (14)	C10—C9—C8	118.75 (16)
O2—N3—C2	118.85 (14)	C10—C9—H9A	120.6
O1—N3—C2	119.18 (13)	C8—C9—H9A	120.6
O4—N4—O3	123.64 (15)	F1B—C10—C11	117.6 (2)
O4—N4—C4	117.97 (15)	F1B—C10—C9	118.2 (2)
O3—N4—C4	118.39 (15)	C11—C10—C9	123.58 (15)
N2—C1—C6	121.26 (14)	C11—C10—H10A	118.2
N2—C1—C2	121.73 (13)	C9—C10—H10A	118.2
C6—C1—C2	117.01 (13)	C10—C11—C12	116.59 (15)
C3—C2—C1	121.42 (14)	C10—C11—H11A	121.7
C3—C2—N3	115.98 (13)	C12—C11—H11A	121.7
C1—C2—N3	122.59 (13)	F1A—C12—C11	116.40 (16)
C4—C3—C2	118.96 (14)	F1A—C12—C13	120.49 (16)
C4—C3—H3A	120.5	C11—C12—C13	123.03 (14)
C2—C3—H3A	120.5	C11—C12—H12A	118.5
C3—C4—C5	121.62 (14)	C13—C12—H12A	118.5
C3—C4—N4	118.27 (14)	C12—C13—C8	119.20 (14)
C5—C4—N4	120.11 (14)	C12—C13—H13A	120.4
C6—C5—C4	119.53 (14)	C8—C13—H13A	120.4
C6—C5—H5A	120.2	C7—C14—H14A	109.5
C4—C5—H5A	120.2	C7—C14—H14B	109.5
C5—C6—C1	121.44 (14)	H14A—C14—H14B	109.5
C5—C6—H6A	119.3	C7—C14—H14C	109.5
C1—C6—H6A	119.3	H14A—C14—H14C	109.5
N1—C7—C8	115.24 (13)	H14B—C14—H14C	109.5
N1—C7—C14	123.22 (14)		
			
C7—N1—N2—C1	−176.16 (14)	C4—C5—C6—C1	0.1 (2)
N1—N2—C1—C6	−0.9 (2)	N2—C1—C6—C5	178.07 (14)
N1—N2—C1—C2	178.42 (13)	C2—C1—C6—C5	−1.3 (2)
N2—C1—C2—C3	−178.00 (14)	N2—N1—C7—C8	−178.27 (12)
C6—C1—C2—C3	1.4 (2)	N2—N1—C7—C14	0.8 (2)
N2—C1—C2—N3	0.7 (2)	N1—C7—C8—C9	170.45 (14)
C6—C1—C2—N3	−179.90 (13)	C14—C7—C8—C9	−8.6 (2)
O2—N3—C2—C3	−4.7 (2)	N1—C7—C8—C13	−8.4 (2)
O1—N3—C2—C3	174.98 (14)	C14—C7—C8—C13	172.56 (15)
O2—N3—C2—C1	176.52 (15)	C13—C8—C9—C10	0.5 (2)
O1—N3—C2—C1	−3.8 (2)	C7—C8—C9—C10	−178.31 (15)
C1—C2—C3—C4	−0.2 (2)	C8—C9—C10—F1B	−171.0 (2)
N3—C2—C3—C4	−178.96 (13)	C8—C9—C10—C11	−0.1 (3)
C2—C3—C4—C5	−1.2 (2)	F1B—C10—C11—C12	170.7 (2)
C2—C3—C4—N4	178.77 (13)	C9—C10—C11—C12	−0.3 (3)
O4—N4—C4—C3	171.96 (16)	C10—C11—C12—F1A	−176.64 (18)
O3—N4—C4—C3	−8.3 (2)	C10—C11—C12—C13	0.1 (3)
O4—N4—C4—C5	−8.1 (2)	F1A—C12—C13—C8	176.95 (17)
O3—N4—C4—C5	171.62 (16)	C11—C12—C13—C8	0.3 (3)
C3—C4—C5—C6	1.3 (2)	C9—C8—C13—C12	−0.6 (2)
N4—C4—C5—C6	−178.70 (14)	C7—C8—C13—C12	178.24 (14)

### Hydrogen-bond geometry (Å, º)

**Table:**

*D*—H···*A*	*D*—H	H···*A*	*D*···*A*	*D*—H···*A*
N2—H1*N*2···O1	0.89 (2)	1.90 (2)	2.6038 (18)	135 (2)
C9—H9*A*···O1^i^	0.93	2.58	3.413 (2)	150
C13—H13*A*···O4^ii^	0.93	2.44	3.176 (2)	137

Symmetry codes: (i) −*x*, *y*+1/2, −*z*+1/2; (ii) −*x*+1, *y*+1/2, −*z*+3/2.
